# Development of the Swahili Cancer Health Literacy Test for use in the African context

**DOI:** 10.4102/phcfm.v18i1.5077

**Published:** 2026-01-10

**Authors:** Kija Malale, Melanie Pienaar

**Affiliations:** 1 Department of Clinical Nursing, School of Nursing, Catholic University of Health and Allied Sciences, Mwanza, Tanzania; 2 School of Nursing, Faculty of Health Sciences, University of the Free State, Bloemfontein, South Africa

**Keywords:** African context, cancer health literacy, health literacy assessment, Swahili health literacy test, Swahili-speaking populations, tool development

## Abstract

**Background:**

As cancer treatment options advance, it is increasingly essential for patients and caregivers to possess adequate cancer-specific health literacy to engage in shared decision-making. While there is growing global interest in developing cultural and disease-specific health literacy tools, Africa remains underrepresented.

**Aim:**

To develop the Swahili Cancer Health Literacy Test (SCHLT) for Swahili-speaking populations in Africa.

**Setting:**

Two settings were involved in developing the SCHLT: Tanzania and South Africa.

**Methods:**

A multimethod design was employed, guided by the MEASURE approach through seven steps: (1) establishing a rationale, (2) creating an empirical framework, (3) developing a theoretical blueprint, (4) constructing an item pool, (5) translating items into Swahili, (6) contextualising the pool and (7) assessing readability. The development process drew on the Integrated Model of Health Literacy (IMHL), the Cancer Control Continuum and the Sesotho Health Literacy Test (SHLT) frameworks.

**Results:**

Nine existing health literacy (HL) tests informed the development of an initial item pool (*n* = 369). Two Delphi rounds achieved consensus on 52 items for the final version. Readability was evaluated using Flesch–Kincaid Grade Level and cognitive interviews with sixth-grade pupils and the target population.

**Conclusion:**

The SCHLT presents a novel, culturally appropriate tool for assessing cancer HL in Swahili-speaking populations. The theoretically grounded development process ensures rigour and provides a model for creating other disease-specific HL tools.

**Contribution:**

This study addresses a significant gap by contributing to the development of cancer HL assessments relevant to African contexts.

## Background

Globally, health literacy is increasingly recognised as essential to cancer care, enabling individuals to navigate the cancer care continuum more effectively.^1,2,3^ The Cancer Control Continuum, which includes aspects such as aetiology, prevention, detection, diagnosis, treatment and survivorship, has become more intricate due to advancements in medical research, technology and treatment options.^4^ As a result, people now face an expanding array of choices and decisions when navigating the services. These complexities have rendered cancer services less accessible and user-friendly for disadvantaged populations, particularly individuals with limited health literacy (HL), whose prevalence ranges from 11.9% to 86% globally.^5^ Studies have shown that individuals with limited HL often face significant challenges in navigating the cancer care system, understanding cancer-related information, adhering to treatment regimens, managing health cost burden and effectively communicating with healthcare providers.^1,2,5,6^ Consequently, they face a disproportionately higher burden of cancer-related premature mortality and diminished quality of life.^1,2,5^ As a global initiative, the World Health Organization (WHO) recently recommended routine HL screening and tailoring cancer-related information in healthcare settings.^7^ While high-income countries have successfully integrated cancer HL screening into routine cancer care, African healthcare systems encounter significant challenges in implementing this initiative, largely due to the lack of a cancer-specific and culturally relevant HL assessment tool. Swahili, a native language, is one of the 10 most spoken languages in the world. It is spoken by over 150 million people in Africa, making it one of the most widely spoken languages on the continent.^8,9^ Despite its broad reach, no culturally relevant, cancer-specific HL assessment tool currently exists for this population.

While general HL tests are widely used, they have been deemed unsuitable for evaluating disease-specific HL, including cancer.^10,11,12,13^ Recently, there has been a significant increase in the development and validation of cancer HL tests globally.^13,14,15,16,17,18,19^ However, most of the tests have been developed and validated in high-income countries, limiting their application in developing countries, especially in Africa. Translation of the tests has been attempted, yet the studies indicate that they are unsuitable, as they either underestimate or overestimate the cancer HL of patients and their caregivers.^16,20^ Other scholars have gone further and considered the translation of the tests without input from local people as an epistemic injustice,^21,22^ underscoring the critical need for a cancer HL test relevant to the African context.

The development of a cancer-specific HL test must be grounded in a strong theoretical framework that defines its content, dimensions, domains and methodological approach. Our initial review of existing frameworks revealed several models related to HL^11,12,23,24^; however, none were specifically tailored to cancer care or aligned with the Cancer Control Continuum, highlighting a critical gap that this study sought to address. The Integrated Model of Health Literacy (IMHL) was found to be prominent across the identified tests, yet not disease specific, resulting in inconsistencies of the construct’s domains, dimensions and core skills across measurements.^12^ To bridge these gaps, the research team integrated insights from the Cancer Control Continuum, the Sesotho Health Literacy Test (SHLT) and the IMHL to guide the systematic development of the Swahili Cancer Health Literacy Test (SCHLT). This study aims to explain the methods and processes employed to develop the context-appropriate SCHLT. By aligning the test with the cancer care continuum and addressing the unique cultural and linguistic needs of Swahili-speaking communities, this methodology provides a replicable framework for other disease-specific tools.

## Research methods and design

### Research design

A multimethod design was employed to develop the SCHLT. Unlike mixed-methods research, which intentionally integrates qualitative and quantitative data, a multi-method design may involve using multiple methods of the same or different types without necessarily merging their data.^25^ The instrument was developed through a structured seven-step process adapted from the MEASURE Approach to Instrument Development.^26^ The MEASURE is an abbreviation that outlines a systematic set of steps: Make the purpose and rationale clear; Establish an empirical framework; Articulate a theoretical blueprint; Synthesise content and scale development; Use expert reviewers; Recruit participants; Evaluate validity and reliability. In this study, the steps were tailored to defining a clear purpose and rationale, establishing an empirical framework, developing a robust theoretical blueprint, synthesising relevant content and scale development, translating and adapting items for contextual relevance, validating item content through expert review, and finally, evaluating the readability of each item to ensure clarity and accessibility. The first four steps utilised resources at the University of the Free State in South Africa, while the final steps took place in Tanzania, where Swahili is widely spoken.

#### Step 1: A clear purpose and rationale

To ensure a clear rationale and purpose, the methodology of a scoping review was used to map the available cancer-specific HL tests for cancer survivors and/or their caregivers. The Joanna Briggs Manual for Evidence Synthesis for conducting a scoping review was used to guide the steps of information retrieval from the selected electronic databases.^27^ After identifying the cancer-specific HL tests, their usability and adaptability were critically evaluated to determine their relevance and applicability within the African context.

#### Step 2: Entailed establishing an empirical framework underpinning the study

To ensure that the development of the SCHLT was theoretically sound, we first reviewed the theoretical frameworks underpinning the cancer HL tests identified in step 1. A suitable theoretical framework was carefully selected and seamlessly integrated with the Cancer Control Continuum model to ensure a comprehensive and contextually grounded approach.

#### Step 3: Entailed articulating the theoretical blueprint of the test

The pool of items from the cancer-specific HL tests identified in Step 1 was iteratively discussed through the brainstorming method by the research team in terms of relevance, and the items were rearranged in the grid according to the established empirical framework in Step 2. Where needed, similar items across the tests were merged. Finally, the items were counted across competencies and domains.

#### Step 4: Involved content synthesis and scale development of the measurement

The item pool generated in Step 1, together with additional items crafted by a multidisciplinary research team comprising nurse oncology practitioners and health communication experts, underwent a rigorous iterative review and refinement process. Guided by the empirical framework from Step 2 and the theoretical blueprint from Step 3, each item was critically examined and rephrased where necessary to align with the cultural and healthcare realities of Swahili-speaking African populations. Through collaborative deliberation, the team reached a unified consensus on the final wording and scaling of all items, ensuring both contextual relevance and conceptual clarity.

#### Step 5: Entailed test item translation

Once the research team reached consensus on the finalised items in Step 4, the process advanced through four systematic translation stages, including preparation, forward translation, back translation and harmonisation.^28^ Two language specialists and two nurse educators independently conducted the forward and back translations, respectively, to ensure both linguistic precision and conceptual equivalence of the items. Both translators involved during forward and backward translations of the items were invited to the harmonisation stage to resolve any discrepancies, thereby ensuring linguistic accuracy and contextual relevance across all items.

#### Step 6: Involved test item content validation

A panel of 17 Swahili-speaking interdisciplinary experts: one medical oncologist, three nurse oncologists, one social worker, one nutritionist, three nurse educators, two language specialists, three palliative care nurses, two cancer survivors and one clinical pharmacist from Tanzania validated the content of the Swahili translated items for clarity, understandability and relevance using a modified contact Delphi technique. All experts were recruited based on predefined criteria outlined by Kalkbrenner,^26^ which included: recognised expertise in the field of oncology, a minimum of 10 years of experience in oncology practice or research and the capacity and willingness to participate in the study. Additionally, fluency in both Swahili and English was mandatory. The workshop was facilitated by three experienced facilitators, two of whom are native Swahili speakers. Delphi round one commenced immediately after obtaining expert consent. The 17 experts were seated in two rows of seven, each provided with a response card labelled ‘Yes’ on one side and ‘No’ on the other to indicate their decisions throughout the session. Clear instructions were given to ensure independent judgement – participants were not allowed to look at, share or discuss their responses with neighbours, except with the facilitators. Each expert received printed copies of all items in both English and Swahili, and every item was simultaneously projected on a PowerPoint screen to maintain focus and consistency. The facilitator guided the process by reading each item aloud and asking whether it was understandable or clear and relevant to the Swahili-speaking context. Experts responded using their cards, and a consensus threshold of 75% or above was used to determine agreement in each category. After completing the first round, participants gathered at a round table during the second round to review and discuss the results. The discussion centred on items that failed to reach the 75% consensus threshold, as well as those that met the threshold in only one domain – clarity or relevance. Experts were encouraged to reconsider their earlier responses in light of the group discussion. Based on these deliberations, the facilitators invited participants to decide whether each contested item should be retained with modifications or omitted entirely. Responses were again indicated using the ‘Yes’ or ‘No’ cards, with a 75% or higher level of agreement required for consensus.

#### Step 7: Entailed the item readability evaluation

The Flesch–Kincaid Grade Level^29^ was employed to evaluate the readability of test items developed in Step 6, as it provides a reliable estimate of the education level required to comprehend short texts, typically sentences or paragraphs under 100 words. A panel of language specialists who are also Swahili native speakers reviewed and amended items found to have a score above Grade 6. In addition, a cognitive interview was conducted with six Grade 6 pupils and five cancer survivors of varying educational levels to assess their understanding of the test items.

### Ethical considerations

Ethical clearance to conduct this study was obtained from the University of the Free State Health Science Research Ethics Committee (No. UFS-HSD2023/2303) and the National Institute for Medical Research (NIMR/HQ/R.8a/Vol. IX/4827) and the Catholic University of Health and Allied Sciences Bugando Research and Ethical Committee (No. CREC/852/2024). Permission to collect data from the participants was obtained from the respective institutions. All participants in steps 5, 6 and 7 were provided with all project details before signing informed consent. An assent form was used instead of a consent form for grade six pupils. Data were collected anonymously and encrypted with password protection, ensuring that only authorised members of the research team can access it. This approach safeguards the confidentiality of all information shared by participants.

## Results

### Step 1: A clear purpose and rationale

As summarised in Table 1, nine cancer-specific HL tests met the inclusion criteria, with the majority developed and validated in high-income countries, particularly the United States. These tests exhibited considerable variability in their underlying frameworks, reflecting inconsistencies in the definition and operationalisation of the cancer HL construct. The dimensionality of the construct (unidimensional versus multidimensional) remains contentious across studies. While dimensions listed in the tests showed minor differences, substantial variation in the domains was evident. Notably, dimensions and domains were often used interchangeably. None of the included tests aligned comprehensively with the conventional Cancer Control Continuum, consisting of aetiology, prevention, detection, diagnosis, treatment and survivorship. Therefore, these limitations necessitated the development of the SCHLT.

**TABLE 1:** Characteristics of the included cancer-specific health literacy measurements (*n* = 9).

Test name	Abbreviation	Number of items	Reference & Country	Dimensionality	Underpinned framework
Chinese version of Cancer Health Literacy Test	CHLT-30-Chinese	30	Chan et al.^14^; China	Unidimensional	Not indicated
	CHLT-6-Chinese	6			
Cancer Health Literacy Scale	C-HLS	33	Chou et al.^15^; Taiwan	Multidimensional	Nutbeam’s 3 constructs of health literacy
Cancer Health Literacy Test	CHLT-30	30	Dumenci et al.^16^; United States	Unidimensional	The continuous latent variable framework
	CHLT-6	6			
Cancer Health Literacy Test-30-Spanish	CHLT-30-DKspa	30	Echeverri et al.^17^; United States	Unidimensional	Not indicated
Assessment of Health Literacy in Cancer Screening	AHL-C	52	Han et al.^30^; United States	Multidimensional	The Institute of Medicine conceptual model of health literacy
Brief generic cancer knowledge scale for patients	BCKS-10	10	Klein et al.^31^; Germany	Multidimensional	Not indicated
Japanese Cancer Intelligence Quotient	JCIQ	34	Minamitani et al.^32^; Japan	Multidimensional	Integrated health literacy model
Assessment of Colon Cancer Literacy	ACCL	10	Pendlimari et al.^33^; United States	Not indicated	Not indicated
Chinese Version of the Breast Cancer Literacy Assessment Tool	C-B-CLAT	10	Shan et al.^34^; China	Multidimensional	Empirical framework

Note: Please see the full reference list of the article Malale K, Pienaar M. Development of the Swahili Cancer Health Literacy Test for use in the African context. Afr J Prm Health Care Fam Med. 2026;18(1), a5077. https://doi.org/10.4102/phcfm.v18i1.5077, for more information

### Step 2: Establishing an empirical framework underpinning the study

**FIGURE 1:**
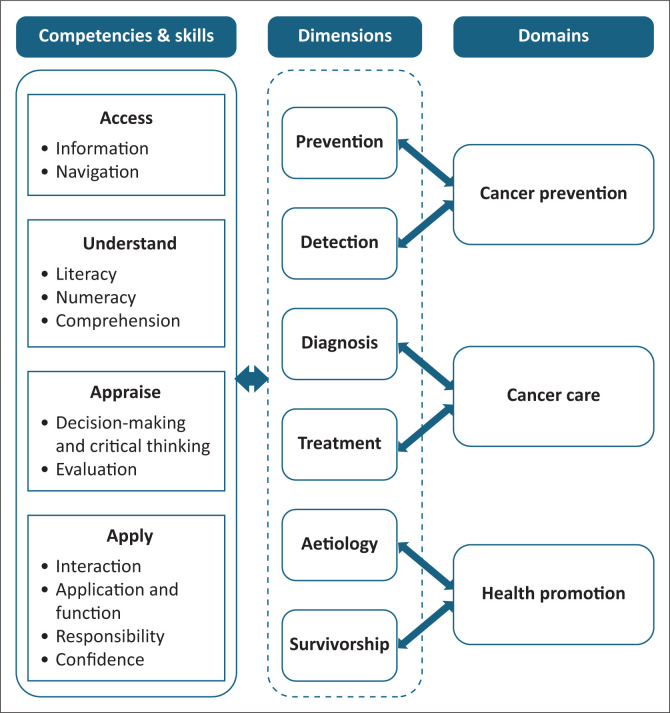
Conceptual framework of the Swahili Cancer Health Literacy Test.

As summarised in Table 1, several theoretical frameworks underpinned the cancer HL tests identified. However, none of the identified frameworks were tailored specifically to the unique complexities and continuum of cancer care. In addition, none of the frameworks integrated the Cancer Control Continuum. To address this situation, the research team synthesised the content of the IMHL, the SHLT’s conceptual framework and the Cancer Control Continuum. As summarised in Figure 1, the IMHL informed the cancer HL domains, encompassing cancer prevention, cancer care and health promotion. These domains are supported by key competencies: accessing, understanding, appraising and applying cancer-related information. The skills derived from the SHLT’s conceptual framework were mapped to the Cancer Control Continuum (aetiology, prevention, detection, diagnosis, treatment and survivorship) as dimensions.

### Step 3: Articulating the theoretical blueprint of the test

Of the 369 test items extracted from the nine cancer HL tests in Step 1, only 111 potentially aligned with the proposed construct. As summarised in Table 2, these items were distributed across three domains, four competencies and nine skills. The majority (86.5%) of the test items focused on cancer care, followed by health promotion (14.4%) and cancer prevention (1%). Among the competencies, understanding accounted for the largest proportion (44.1%), followed by applying (24.3%), appraising (18.0%) and accessing (13.5%). However, the distribution of items across specific skills was uneven, with information (2 items), navigation (16 items), numeracy (10 items), comprehension (36 items), decision-making or critical thinking (8 items), evaluation (19 items), interaction (7 items), responsibility (5 items) and confidence (8 items). Notably, no items were identified for literacy and applicability skills.

**TABLE 2:** Theoretical blueprint of items for the Swahili Cancer Health Literacy Test (*n* = 111).

Skills	Dimension and competencies												Total
	Cancer promotion				Cancer prevention				Cancer care				
	Access	Understand	Appraise	Apply	Access	Understand	Appraise	Apply	Access	Understand	Appraise	Apply	
Information†	-	-	-	-	-	-	-	-	2	-	-	-	**2**
Navigation†	-	-	-	-	-	-	-	-	13	3	-	-	**16**
Literacy‡	-	-	-	-	-	-	-	-	-	-	-	-	**0**
Numeracy‡	-	3	-	-	-	-	-	-	-	7	-	-	**10**
Comprehension‡	-	-	-	-	-	1	-	-	-	35	-	-	**36**
Decision-making or critical thinking§	-	-	-	-	-	-	-	-	-	-	1	7	**8**
Evaluation§	-	-	2	-	-	-	-	-	-	-	17	-	**19**
Interaction¶	-	-	-	4	-	-	-	-	-	-	-	3	**7**
Application and function¶	-	-	-	-	-	-	-	-	-	-	-	-	**0**
Responsibility¶	-	-	-	3	-	-	-	-	-	-	-	2	**5**
Confidence¶	-	-	-	4	-	-	-	-	-	-	-	4	**8**

**Total**	**0**	**3**	**2**	**11**	**-**	**1**	**0**	**0**	**15**	**45**	**18**	**16**	**111**

† , Skills for accessing health information competencies;

‡ , Skills for understanding health information competencies;

§ , Skills for appraising health information competencies;

¶ , Skills for applying health information competencies.

### Step 4: Content synthesis and scale development of the measurement

**FIGURE 2:**
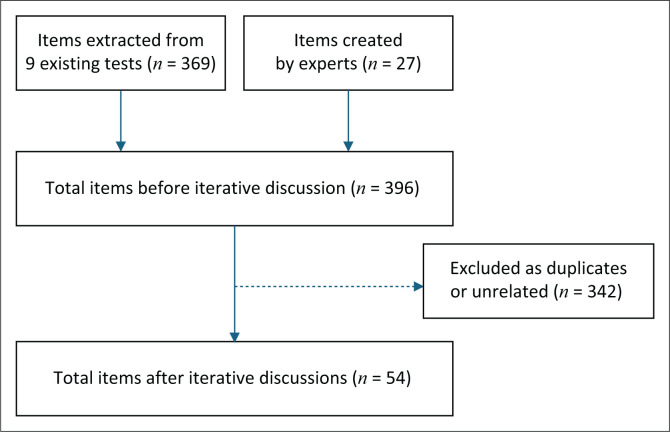
Work floor of content synthesis for the Swahili Cancer Health Literacy Test.

As summarised in Figure 2, of the 396 initial test items (369 extracted from existing cancer-specific HL tests and 27 newly developed by the experts), iterative reviews eliminated 342 items due to redundancy or irrelevance to the proposed construct. Fifty-four items were considered eligible for further evaluation. Based on the experience of the research team, the Guttman scale^11,35^ consisting of ‘Yes’, ‘No’ or ‘I don’t know’ to each item was considered relevant and applied to the current test.

### Step 5: Test item translation

The test items from Step 3 were translated into Swahili, back-translated and harmonised as per the recommended four systematic stages.^28^

### Step 6: Test item content validation

Of the 54 items presented to the panel of interdisciplinary experts, 49 (90.7%) were deemed relevant to the proposed test during the first round of the Delphi session, increasing to 96.3% in the second round of the Delphi session. Among the 19 items that scored less than 75% in both understandability or clarity and relevancy or 75% or higher in either category during round one, 17 items (89.5%) were retained and revised during round two. Ultimately, the panel of experts affirmed 52 items as understandable, clear and relevant for the SCHLT (Appendix 1, Table 1-A1).

### Step 7: Swahili Cancer Health Literacy Test items readability evaluation

Flesch–Kincaid Grade-Level scores of test items ranged from 12.30 to 20.88 due to Swahili’s syllable-rich structure. Cognitive interviews with grade 6 pupils and cancer survivors confirmed the understandability and appropriateness of the test items for individuals with primary education levels.

## Discussion

Health literacy is a complex and multidimensional construct that requires disease-specific and contextually relevant measurement tools to enable healthcare providers to identify individuals with limited HL and deliver tailored interventions effectively. This study conceptualised and developed the SCHLT, a cancer-specific HL test designed for Swahili-speaking African populations. The development process highlighted the inadequacy of existing cancer HL instruments in capturing the unique contextual and linguistic realities of African settings, as well as the absence of comprehensive theoretical frameworks aligned with the Cancer Control Continuum. It also revealed inconsistencies in item distribution across domains, competencies and dimensions and the inapplicability of conventional English readability assessments due to linguistic differences. A key insight from this process is that building a robust, disease-specific HL tool demands a systematic and evidence-driven approach that integrates a clear conceptual rationale, a strong empirical and theoretical foundation, culturally attuned item development, meticulous translation and thoughtful contextual adaptation to ensure both linguistic precision and conceptual clarity. This rigorous, stepwise methodology not only strengthens the validity and reliability of the SCHLT but also offers a replicable framework for developing other disease-specific HL instruments in low-resource contexts. Despite certain limitations, this study provides a foundational contribution to advancing cancer HL research and practice in Africa.

An instrument development study is necessary if the literature lacks a test to appraise the researcher’s desired construct, if the existing instrument is potentially psychometrically flawed, or inappropriate for use with their target population. For the SCHLT, a scoping review identified several cancer-specific HL measurements; however, most were unsuitable for African populations, as they were primarily developed in high-income countries with significantly different cultural and economic contexts. Similar observations were reported in other studies when developing general HL measurements.^11,36,37^ Although translating existing tests is recommended, recent studies indicate that translation alone does not guarantee their relevance to the target country’s health system, health concepts or cultural beliefs.^11^ This gap warranted the development of the SCHLT for more than 150 million Swahili-speaking people in Africa. The test is designed to assist healthcare professionals in cancer care facilities by identifying individuals with limited cancer HL. In this way, interventions may be tailored to the unique needs of the patient, ultimately enhancing their participation in shared decision-making within the complex cancer care system.

Developing a disease-specific HL test requires a comprehensive theoretical framework aligned with the disease care continuum to provide a series of principles or assumptions that underline the proposed measurement construct.^26,38^ Achieving this step requires thoroughly reviewing existing theoretical frameworks to identify their relevance for the intended test.^26^ In the case of SCHLT, a thorough review of the theoretical frameworks underpinning the cancer HL measurements was conducted. However, none of the identified theoretical frameworks focused on specific diseases, such as cancer. The broad nature of existing frameworks contributed to inconsistencies in the construct domains, dimensions and dimensionality across various tests. Importantly, none of them aligned with the conventional Cancer Control Continuum.^4^ The research team integrated elements from multiple frameworks, including the IMHL,^12^ the SHLT Conceptual Framework^11^ and the National Cancer Institute Cancer Control Continuum,^4^ to bridge this gap. The IMHL informed the construct domains and competencies. The domains encompass cancer prevention, cancer care and health promotion, each reinforced by the core competencies of accessing, understanding, appraising and applying cancer-related information to promote informed choices and optimal health outcomes. The National Cancer Institute’s Cancer Control Continuum informed the skills’ dimensions, including aetiology, prevention, detection, diagnosis, treatment and survivorship. The SHLT conceptual framework informed the skills across the competencies. Unlike previous frameworks, the current framework is specifically designed for cancer HL but can be adapted for other diseases by integrating their respective care continuum into the skills dimension.

Articulating a theoretical blueprint helps developers ensure the content validity of a test when creating the content and domain areas for the construct and determining the approximate proportion of items that should be developed across each content and domain area.^26^ It is established by aligning items from existing assessments. The grid table detailing skills, competencies, dimensions and domains from existing cancer HL literacy measurements served as a theoretical blueprint for the SCHLT. However, the test items from previous cancer HL tests were unevenly distributed across skills, competencies, dimensions and domains, necessitating the development of new test items. This distribution highlights the emphasis on cancer care and educational interventions aimed at addressing challenges encountered by patients and their caregivers in navigating the cancer care system.^7^

A systematic approach to test item synthesis and scale development is crucial for ensuring the test’s theoretical, contextual and disease-specific validity and reliability. Unlike the approach used in the previous tests, researchers in the current study primarily extracted and iteratively refined items from existing cancer-specific HL tests based on an established empirical framework. In addition, this study incorporated expert-driven item construction and rigorous content validation. This methodology strengthened the robustness of the process, ensuring a more comprehensive and contextually relevant test. Furthermore, utilising the Guttman scaling format, a common approach in test development, enhances the test’s simplicity and effectiveness in evaluating respondents’ cancer HL.^11,35^

Ensuring accurate translation and readability of the test items is crucial in contextualisation and usability of the proposed test. In the case of SCHLT, the effectiveness of forward and backward translation, followed by harmonisation to resolve discrepancies, was confirmed. This study also identified the inadequacy of readability evaluation tests for English texts due to linguistic differences, particularly the higher syllable count per sentence in Swahili. Similar challenges have been reported in other studies. For example, Krige et al.^39^ observed variations in readability scores when assessing Sesotho-written pamphlets in South Africa using Flesch Reading Ease, Flesch–Kincaid, Fry and Coleman–Liau Index determinations. The authors observed high readability scores in Sesotho-written sentences due to structural differences, as Sesotho contains many monosyllabic and single-letter words. To address this limitation, the current study employed alternative approaches, including cognitive interviews with grade 6 pupils and a sample of the targeted population. These interviews ensured that all test items were easily understood, regardless of participants’ educational backgrounds, reinforcing the suitability of the assessment tool.

Despite the methodological robustness employed during the development of the SCHLT, several limitations should be acknowledged. Firstly, the study primarily focused on Swahili-speaking populations, which may limit its applicability to other linguistic and cultural groups within Africa. Future research should focus on adapting and validating the test across countries with varying Swahili pronunciations to ensure its linguistic accuracy, cultural relevance and broader applicability across Swahili-speaking populations. Secondly, while rigorous methods were employed in item selection, content validation and readability assessment, the study did not assess the tool’s predictive validity or effectiveness in measuring cancer HL over time. Longitudinal studies are needed to evaluate how well the test predicts health outcomes and patient engagement in cancer care. Thirdly, the study used cognitive interviews with grade 6 pupils and cancer survivors to assess readability and comprehension. Although this approach ensured clarity, it may not fully capture the perspectives of all individuals with low literacy levels, particularly older adults and those with minimal formal education. Future studies should consider a broader demographic representation during validation. Fourthly, while expert reviews were conducted to refine the test, the lack of a large-scale field validation study means that further research is needed to confirm its reliability and validity across diverse healthcare settings. Conducting large-scale psychometric evaluations will strengthen the test’s robustness and applicability.

## Conclusion

This study has showcased the conceptualisation and development of a disease-specific HL test, the SCHLT for African Swahili-speaking populations. The development of the SCHLT addressed existing methodological gaps and enhanced cultural and linguistic appropriateness by establishing a clear rationale, creating a disease-specific empirical framework, developing a theoretical blueprint, constructing an item pool, translating items into Swahili, contextualising the item pool and ensuring item readability. This tailored methodology strengthens the rigour of the test and may be applied to other disease-specific HL tests. While the study has certain limitations, it lays a critical foundation for future research on cancer HL in resource-limited settings. Further validation and large-scale implementation will strengthen its applicability and inform targeted interventions that improve cancer awareness, early detection and patient engagement in cancer care. Ultimately, this test has the potential to contribute significantly to health policy reforms and cancer control strategies in Africa.

## Appendix 1

**Table 1-A1:** Output summary of the modified delphi rounds.

Contact Delphi round	Output
Round one	Items with a consensus score of ≥ 75% both in understandability/clarity and relevancy (*n* = 35): 1, 4, 7, 8, 9, 10, 11, 13, 14, 15, 16, 17, 18, 19, 20, 21, 22, 23, 28, 30, 33, 34, 37, 38, 41, 42, 43, 45, 46, 47, 49, 50, 51, 53, 54
	Items with a consensus score of ≥ 75% in understandability/clarity only (*n* = 2): 39, 40
	Items with a consensus score of ≥ 75% in relevancy only (*n* = 14): 2, 3, 5, 6, 12, 25, 26, 27, 29, 31, 32, 35, 36, 52
	Items with a consensus score of < 75% both in understandability/clarity and relevancy (*n* = 3): 24, 44, 48
Round two	Items with a consensus score of ≥ 75% both in understandability/clarity and relevancy (*n* = 35): 1, 4, 7, 8, 9, 10, 11, 13, 14, 15, 16, 17, 18, 19, 20, 21, 22, 23, 28, 30, 33, 34, 37, 38, 41, 42, 43, 45, 46, 47, 49, 50, 51, 53, 54 were all retained.
	Items with a consensus score of ≥ 75% in understandability/clarity only (*n* = 2): 39, 40 were amended, and all retained.
	Items with a consensus score of ≥ 75% in relevancy only (*n* = 14): 2, 3, 5, 6, 12, 25, 26, 27, 29, 31, 32, 35, 36, 52 were amended and all retained.
	Items with a consensus score of < 75% both in understandability/clarity and relevancy (*n* = 3): 24, 44, 48. Only item number 44 was amended and retained.
